# Assessing the usability of wearable devices to measure gait and physical activity in chronic conditions: a systematic review

**DOI:** 10.1186/s12984-021-00931-2

**Published:** 2021-09-15

**Authors:** Alison Keogh, Rob Argent, Amy Anderson, Brian Caulfield, William Johnston

**Affiliations:** 1 UCD School of Public Health, Physiotherapy and Sports Science, University College Dublin, Dublin, Ireland; 2 Insight Centre for Data Analytics, University College Dublin, Dublin, Ireland; 3 Health Service Executive, Dublin, Ireland

**Keywords:** Usability, Wearable sensors, Gait, Physical activity, User experience

## Abstract

**Background:**

The World Health Organisation’s global strategy for digital health emphasises the importance of patient involvement. Understanding the usability and acceptability of wearable devices is a core component of this. However, usability assessments to date have focused predominantly on healthy adults. There is a need to understand the patient perspective of wearable devices in participants with chronic health conditions.

**Methods:**

A systematic review was conducted to identify any study design that included a usability assessment of wearable devices to measure mobility, through gait and physical activity, within five cohorts with chronic conditions (Parkinson’s disease [PD], multiple sclerosis [MS], congestive heart failure, [CHF], chronic obstructive pulmonary disorder [COPD], and proximal femoral fracture [PFF]).

**Results:**

Thirty-seven studies were identified. Substantial heterogeneity in the quality of reporting, the methods used to assess usability, the devices used, and the aims of the studies precluded any meaningful comparisons. Questionnaires were used in the majority of studies (70.3%; n = 26) with a reliance on intervention specific measures (n = 16; 61.5%). For those who used interviews (n = 17; 45.9%), no topic guides were provided, while methods of analysis were not reported in over a third of studies (n = 6; 35.3%).

**Conclusion:**

Usability of wearable devices is a poorly measured and reported variable in chronic health conditions. Although the heterogeneity in how these devices are implemented implies acceptance, the patient voice should not be assumed. In the absence of being able to make specific usability conclusions, the results of this review instead recommends that future research needs to: (1) Conduct usability assessments as standard, irrespective of the cohort under investigation or the type of study undertaken. (2) Adhere to basic reporting standards (e.g. COREQ) including the basic details of the study. Full copies of any questionnaires and interview guides should be supplied through supplemental files. (3) Utilise mixed methods research to gather a more comprehensive understanding of usability than either qualitative or quantitative research alone will provide. (4) Use previously validated questionnaires alongside any intervention specific measures.

**Supplementary Information:**

The online version contains supplementary material available at 10.1186/s12984-021-00931-2.

## Background

Healthcare research is in the midst of a paradigm shift with a move towards more long-term behavioural monitoring through the use of wearable devices. This shift has been recognised by the World Health Organisation (WHO) through its recent publication of a digital health strategy [1]. While wearable devices offer researchers access to previously unattainable information regarding how people behave, additional factors need to be considered when designing and implementing these devices including patient safety, privacy, cost-effectiveness etc. Of critical importance, is that this digital shift should be patient-centred, evidence based, inclusive and contextualised [1].

Whether a device is considered usable by the person who will be wearing it has been identified as “among the most important considerations with patient-orientated digital-based solutions” [2]. The International Organization for Standardization defines usability as “the effectiveness, efficiency, and satisfaction with which specified users achieve specified goals in particular environments” [3]. In this manner usability is a broad concept that can also include the acceptability of, or satisfaction with, a device, while the WHO lists the evaluation of the usability and feasibility of a device being the first steps that should be undertaken when assessing any new digital health intervention [1, 4]. It has been suggested that for wearable devices to be accepted, they must be easy to wear, easy to use, affordable, contain relevant functionality and be aesthetically pleasing [5–8]. Usability, by its nature, is context specific and understanding how context may influence adoption has been highlighted as a research need in this area [9]. The acceptability of the above will depend on the length of time the device needs to be worn for and the characteristics of those using it, including their health conditions. The concept of usability is therefore almost never ending, as researchers and digital health developers need to ensure that their selected device is fit for purpose within all aspects of their study design. Failure to assess usability may result in researchers implementing devices that are not worn, that are worn or used incorrectly, and thus may negatively impact data collection and quality and limit the impact of any intervention [1, 10, 11].

Usability is likely to be specifically important in contexts where wearable devices are designed to be implemented during real-world tasks or activities. Walking (including gait and physical activity) in particular, is a functional task that is part of most activities of daily living and has been identified as so critical to health, that is has been labelled a ‘vital sign’ [12, 13]. Many consumer wearable measure activity as standard, but in-depth gait analysis, and the production of digital biomarkers linked to gait, is becoming an important feature of current and future research as it is recognised that understanding how people move can inform researchers and clinicians alike of patient progress, behaviour change and intervention effectiveness [14–17]. Thus, if walking is a key activity being measured by wearables, it is important to understand how usable these devices are in this context. To date, most usability studies have evaluated devices in healthy adults, or have focused primarily on consumer and/or watch based devices [5, 18–25]. However, the needs of healthy adults are likely to be very different than those with chronic health conditions with which many of these devices are deployed to support. For example, issues with fine motor control, skin sensitivity, or balance deficits may be present in clinical cohorts and may be aggravated by the use of certain devices depending on their size, materials and interactivity. A 2015 study suggested that wearable devices are generally accepted by people with chronic conditions, however this research failed to report what type of wearables were assessed, or what chronic conditions were included in the analysis [20]. It has been suggested that health conditions and the specific measurement needs of conditions impacts participant adherence [21]. Specifically in relation to walking, many chronic conditions are associated with symptoms that may impact how well an individual can walk, but the pathophysiology’s and the impact on mobility may be very different [15], for example cardiorespiratory conditions vs neurological. Wearable devices need to be usable across a comprehensive trajectory of mobility problems, thus it is worth exploring users perceptions across multiple cohorts so as to broadly determine their usability in people with chronic conditions. Therefore, this review focused on five clinical cohorts, specifically respiratory problems (chronic obstructive pulmonary disease—COPD), neurodegenerative conditions (Parkinson’s disease—PD), neuroinflammatory problems (multiple sclerosis—MS), osteoporosis and sarcopenia (hip fracture recovery/proximal femoral fracture—PFF), and cardiac pathology (congestive heart failure—CHF). Combined these cohorts are highly prevalent conditions with significant associated disability. Specifically, COPD is the most prevalent chronic respiratory illness globally [26], the rate of prevalence and burden for MS and PD are growing and for PD have doubled [27, 28], PFF is the fracture with the greatest direct cost to the community [29], CHF accounts for up to 2% of healthcare expenditure, while all conditions are associated with greater falls risk which are a significant cause of death and disability globally [30]. Collectively these conditions represent broad array of mobility problems with different trajectories of disability, thus allowing for a comprehensive evaluation of mobility. Although it is likely that some differences in usability may be noted between cohorts, it is nonetheless worth comparing across common conditions to determine where differences and similarities in usability exist.

To the author’s knowledge no systematic review investigating the usability of wearable devices specifically for mobility exists, Given the dearth of literature examining the usability of wearable devices for the assessment of walking in cohorts with chronic health conditions, further evaluations of this are required to support the use and development of these devices in the future. Therefore, this study aimed to conduct a systematic review of the literature to explore the usability of wearable devices to monitor gait and activity in five common patient cohorts where digital mobility assessment may be clinically useful to monitor their symptoms and progress.

## Methods

### Protocol

This study was pre-registered on PROSPERO (ID: CRD42020165301) and was performed in accordance with the PRISMA (Preferred Reporting Items for Systematic Reviews and Meta-Analysis) statement [31].

### Search strategy and eligibility criteria

In January 2020 a search strategy was implemented within PubMed, EMBASE, Medline and Cinhal Plus. The available literature was systematically searched for studies of any research design, that assessed the usability of wearable devices to measure gait or physical activity, in any of the clinical cohorts in Mobilise-D. No language restrictions (including time and language) were applied in any of the databases, while publication dates were open ended up to the search date of January 31st 2020. The strategy was adapted for each database (Table 1).

**Table 1:** Search strategy

Component	String
Participants	1. Parkinson’s OR Parkinson’s disease OR Parkinsonism or Parkinsonian
	2. Multiple sclerosis OR demyelinating disease
	3. Proximal femoral fracture OR femoral fracture OR femur fracture OR hip fracture ORtrochanteric fracture
	4. Chronic obstructive pulmonary disease OR chronic airflow obstruction OR chronic obstructive airway disease
	5. Congestive heart failure OR congestive cardiac failure OR myocardial failure OR heart failure
	6. 1 OR 2 OR 3 OR 4 OR 5
Intervention	7. Wearable electronic device OR inertial measurement unit OR wearable sensor OR activity tracker OR accelerometer OR gyroscope OR magnetometer
Outcome	8. Usability OR acceptability OR human computer interface OR patient satisfaction OR questionnaire OR interview OR self-report
	6 AND 7 AND 8

Two authors (AK and AA) reviewed all titles and abstracts and obtained the full texts of potentially eligible studies. Following this, full-text were independently assessed for eligibility. In instances of disagreement, a third author (WJ) was included for consensus. Studies were included if they fulfilled the criteria outlined within Table 2.

**Table 2:** Inclusion and exclusion criteria

Inclusion criteria
Deploy a wearable sensor to measure gait or physical activity for any duration of time
Any one of the following clinical cohorts
Parkinson’s Disease
Multiple Sclerosis
Congestive Heart Failure
Chronic Obstructive Pulmonary Disorder
Proximal Femoral Fracture
included terms such as:
Acceptability
Wearability
Perceived usefulness
Perceived ease of use
Perceived comfort

Exclusion criteria
No wearable sensor worn or sensors which did not measure gait or physical activity
Any study design
Must have assessed usability, as determined by the participant, in some manner, either qualitatively or quantitatively. Usability
Not including one of the identified clinical cohorts
No usability assessment completed, or the assessment did not consider the participant’s opinion

### Data extraction

Two authors (AK and RA) independently extracted information regarding the assessment of usability, using a piloted data extraction form. Specifically, the method of assessment (i.e. whether it was qualitative or quantitative) was noted, alongside the factors that were evaluated as part of the usability assessment, the method of analysis used, the name of the questionnaire used (if applicable), and the main findings in relation to the usability assessment. In addition, the following data were also extracted: study design and aim, number of participants, participant characteristics, number of devices used, the anatomical location they were worn, how they were attached to the participant, the duration of their use, whether participant engagement with the device was required, whether the wearable was linked to an additional device, and the context in which the device was deployed (i.e. remote or in a laboratory/clinic environment).

The quality of the included texts was evaluated by two authors (AK and AA). To determine the quality of reporting in studies involving qualitative research, the COREQ checklist was used [32]. COREQ is 32-item a reporting guideline checklist for interviews and focus group. Although not an appraisal tool it may acts as a method to judge reporting quality. The authors (AK and RA) determined whether each of the 32-items had been reported or not. The AXIS tool is a critical appraisal tool of quality for cross-sectional studies [33], which contains 20 questions to assess quality. The authors (AK and AA) responded to each question using yes (+), no (−) or don’t know (?) to judge overall quality with the AXIS tool. Quality of reporting is judged subjectively, with no clear criteria as to what constitutes high or low quality.

### Analysis

A narrative synthesis of the data was completed by reporting the findings related to the study characteristics, wearable devices and systems, usability assessment (quantitative methods), usability assessment (qualitative methods) and study quality. Due to the heterogeneity of the data extracted from the included studies, no formal statistical analysis or meta-analysis was possible. Therefore, the results of this review are listed descriptively.

## Results

**Fig. 1:**
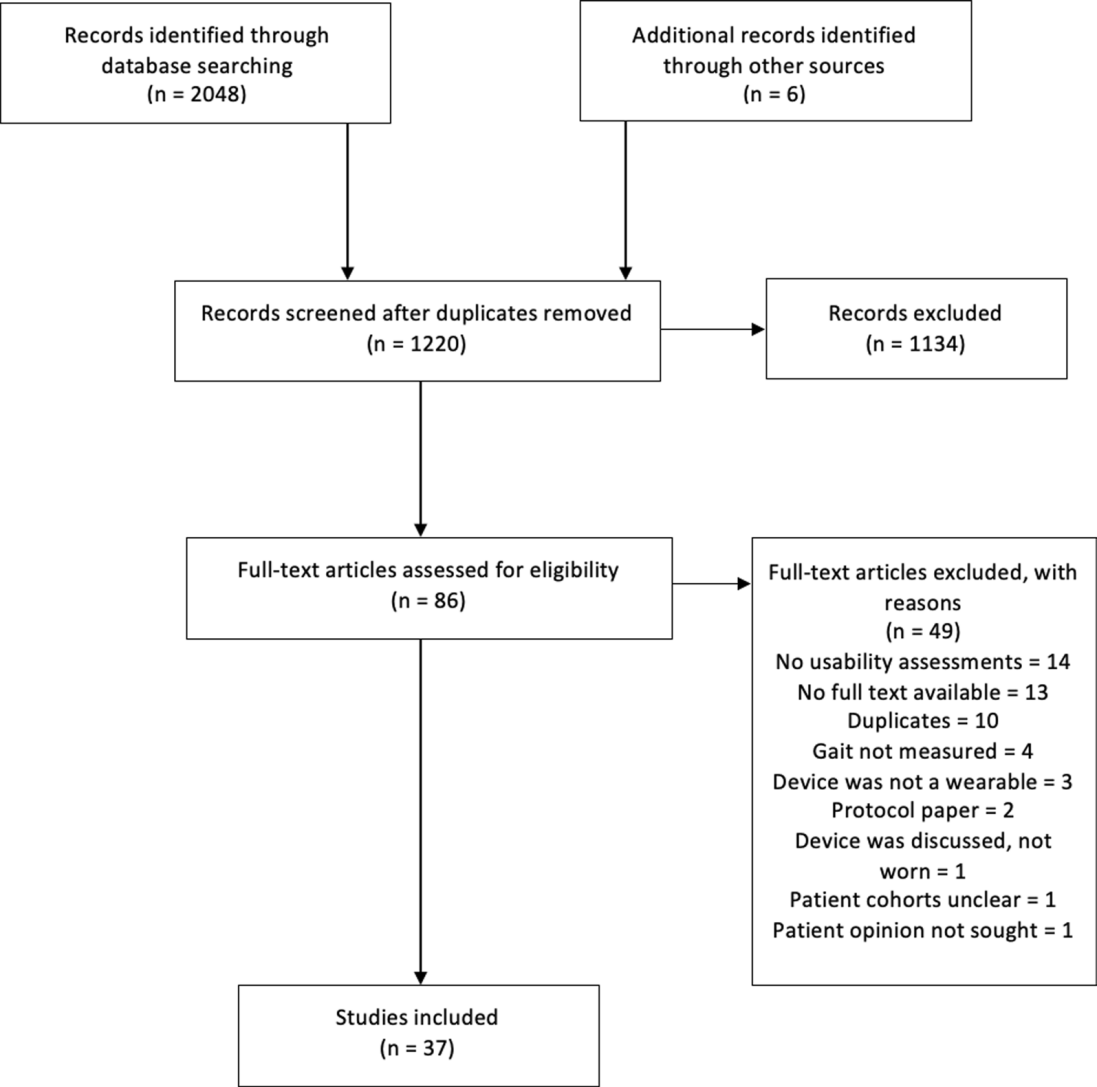
Flowchart of included studies

A total of 2054 articles were identified, of which 834 were duplicates. Following exclusion (reasons listed in Fig. 1) a total of 86 studies were selected for full-text screening, of which 37 were included for analysis [34–70].

### Study characteristics

The characteristics of the studies are displayed in Table 3. Year of publication ranged from 2008 to 2019. The majority of studies were cross-sectional (83.8%; n = 31). Studies reported a variety of aims, which were categorised as: (i) assessing the usability of a wearable device/system (24.3%, n = 9), (ii) presenting or describing the development of a wearable device/system (8.1%, n = 3), (iii) assessing the feasibility and acceptability of a wearable device/system (59.5%, n = 22), and (iv) interventions to alter participants behaviour (8.1%, n = 3).

**Table 3:** Participant information

	Number of participants Mean (SD)	Age of participants Mean (SD)	Percentage of male participants Mean % (SD)	Average year with condition
Clinical cohorts (n = number of studies)				
Overall (n = 37)	24.0 (2.8)	75.5 (3.5)	71.4% (19.3)	8.3 (3.4)
PD (n = 18)	70.3 (196.5)	65.5 (7.6)	61.8% (12.3)	7.6 (3.0)
MS (n = 4)	36.0 (27.8)	57.6 (15.8)	18.0% (15.6)	9.2 (3.0)
CHF (n = 6)	15.0 (10.9)	61.3 (3.5)	56.1% (6.8)	NR
COPD (n = 9)	51.2 (77.0)	61.2 (10.0)	63.4% (21.2)	NR
Healthy volunteers (n = number of studies)				
Overall (n = 5)	28.0 (23.5)	50.4 (21.3)	49.4% (28.3)	N/A
PD (n = 2)	15.0 (N/A)	62.2 (5.9)	45.0% (21.2)	N/A
MS (n = 1)	25.0 (N/A)	34.9 (N/A)	72.0 (N/A)	N/A
CHF (n = 0)	N/A	N/A	N/A	N/A
COPD (n = 2)	36.0 (37.8)	46.3 (35.0)	42.5% (46.0)	N/A

Almost half of the studies were completed in participants with PD (n = 18; 48.6%). COPD accounted for 24.3% of studies (n = 9), CHF was studied in 16.2% (n = 6) while MS was assessed in 10.8% of studies (n = 4). No study assessed PFF. On average, 24 participants (2.8) wore the wearable devices within the included articles (Table 3).

### Wearable devices and systems

The majority of studies implemented a single device to measure gait or physical activity (n = 19; 51.4%). Across the 37 studies, 32 different wearable devices or systems were deployed, thus limiting the comparisons between them.

Devices were attached to 11 different anatomical sites on the body (Table 4), of which the wrist was the most common site (43.2%; n = 16) [37–39, 41, 43, 49–51, 54, 56–58, 61, 69–71], followed by the waist or lower back (27.1%; n = 10) [36, 38–40, 46, 55, 56, 58, 61, 67]. With regards to the method of attachment, eight different methods were used (Table 4) of which straps (45.9%; n = 17) [35–41, 43, 46, 47, 49, 54, 57–61, 70] and clips were the most commonly used (16.2%; n = 6) [45, 52, 56, 62, 64, 65]. Nine studies failed to report how the wearable was attached to the body (24.3%) [42, 45, 48, 50, 51, 55, 63, 68, 69], while four failed to report where they were attached (10.8%) [42, 63, 65, 68].

**Table 4:** Overview of all included studies, the type and number of devices worn, the duration of their use and the method of usability assessment

Author	Year	Condition	Device name	No. worn	Method of attachment	Location of attachment	Context	Duration of use (days)	Method of assessment
Adams et al.	2017	PD	Mc10 Biostamp	5	Adhesive	Chest, forearms, thighs	Lab plus remote	2	Questionnaire
Albani et al.	2019	PD	Custom device: upper limb camera & glove combined with Shimmers	4	Strap/Gloves	Chest, hands, thighs	Lab/Clinic	NR	Questionnaire
Bachlin et al.	2010	PD	NR	3	Strap	Waist, thigh, shins	Lab/Clinic	NR	Questionnaire
Botros et al.	2019	PD	Axivity	3	Strap	Wrist, hip, ankle	Remote	28	Questionnaire
Cancela et al.	2014	PD	Custom IMU system	5	Strap	Wrist, shins, waist	Remote	6	Interviews
Cancela et al.	2014	PD	Custom IMU system	5	Strap	Wrist, shins, waist	NR	NR	Mixed methods
Carpinella et al.	2017	PD	Gamepad using TMA sensors	6	Strap	Thigh, shins, waist, chest	Lab/Clinic	> 1	Questionnaire
Chiauzzi et al.	2019	MS	Fitbit	1	Strap	Wrist	Remote	17	Interviews
Colon-Semenza et al.	2017	PD	Fitbit Zip	1	NR	NR	Remote	32	Mixed methods
Deka et al.	2018	CHF	Fitbit Charge HR	1	Strap	Wrist	Remote	32	Questionnaire
Ellis et al.	2013	PD	Omron HJ- 720ITC pedometer	1	Clip	Hip	Remote	30	Questionnaire
Ellis et al.	2018	PD	Fitbit Zip	1	NR	Hip	Remote	365	Questionnaire
Ferreira et al.	2015	PD	NR	3	Strap	Wrist, leg, waist	Remote	84	Mixed methods
Floegel et al.	2019	CHF	Tractivity	3	Strap	Chest, thigh, ankle	Remote	30	Interviews
Ginis et al.	2016	PD	EXLs3	2	NR	Feet	Remote	42	Questionnaire
Heijmans et al.	2019	PD	MOX5,	3	Strap	Wrist, chest	Remote	14	Questionnaire
Hermanns et al.	2019	PD	Fitbit Alta HR	1	NR	Wrist	Remote	84	Mixed methods
Joshi et al.	2019	PD	Personal KinetiGraph (PKG)	1	NR	Wrist	Remote	6	Questionnaire
KaY et al.	2008	MS	Actical accelerometer	2	Clip	Hip	Lab/Clinic	> 1	Questionnaire
McNamara et al.	2016	COPD	SenseWear Armband	1	Armband	Arm	Remote	7	Questionnaire
Midaglia et al.	2019	MS	Floodlight system	2	Strap/pouch	Wrist, hip	Remote	168	Questionnaire
Moy et al.	2012	COPD	Omron HJ- 720ITC pedometer	1	NR	Waist/Lower back	Remote	90	Interviews
Orme et al.	2018	COPD	Lumo; Actigraph	2	Clip	Wrist, waist	Remote	14	Interviews
Silva de Lima et al.	2017	PD	Fox Wearable Companion smartwatch (Pebble) with smartphone	1	Strap	Wrist	Remote	70	Questionnaire
Stack et al.	2016	PD	Non-commercially available devices	5	Strap	Wrist, ankle, waist	Lab/Clinic	> 1	Interviews
Strisland et al.	2013	CHF	ESUMS Wearable Device	1	Strap	Chest	Remote	14	NR
Svagard et al.	2014	CHF	ESUMS Wearable Device	1	Strap	Chest	Remote	14	Mixed methods
Tzallas et al.	2014	PD	PERFORM system; ALA-6 g	5	Strap	Wrist, ankle, waist	Remote	5	NR
van der Weegen et al.	2014	COPD	NR (it's LiFe tool)	1	Clip	Hip	Lab plus remote	84	Mixed methods
Varnfield et al.	2011	CHF	Nkia N96 smartphone	1	NR	NR	Remote	42	Mixed methods
Verwey et al.	2014	COPD	MOX activity monitor	1	Clip	Hip	Remote	84	Interviews
Verwey et al.	2016	COPD	MOX activity monitor; it's LiFe tool	1	Clip	NR	Remote	84	Mixed methods
Vooijs et al.	2014	COPD	Fitbit Ultra; Personal Activity Monitor AM300	1	In pocket	Hip	Remote	7	Questionnaire
Vorrink et al.	2016	COPD	HTC Desire A8181 mobile phone; BHC0100 Sensewear PRO armband	2	Armband, pouch	Waist, arm	Remote	21	Mixed methods
Wendrich et al.	2019	MS	Fitbit Charge 2	1	NR	NR	Remote	28	Interviews
Werhahn et al.	2019	CHF	iPhone 6Se and apple Watch 1st gen	2	NR	Wrist	Remote	56	Questionnaire
Wu et al.	2018	COPD	Android watch	1	Strap	Wrist	Remote	90	Interviews

The majority of participants were asked to wear their devices remotely (n = 29 studies; 78.4%) [34, 37, 38, 41–51, 53–57, 59–70]. On average, participants were asked to use devices for 203.5 days (228.4). Anything from 48 h to 12 months was reported in studies (Table 4), however the majority of studies asked participants to utilise devices for 7 days or longer (n = 27; 73.0%) [37, 41–50, 53–57, 59, 60, 62–70].

Most wearables were used as part of a monitoring system (n = 32; 86.5%) [36–38, 40–50, 52, 54–70], often where there was a requirement to link devices to either a smartphone/tablet with an app (n = 23; 62.2%) [38, 42–46, 48–51, 54, 56, 57, 59–70]. Required engagement from participants was poorly reported and was unclear or not reported in 45.9% of studies (n = 17) [34, 35, 37, 38, 40, 47, 49, 51–53, 56–58, 60, 61, 66, 67]. However when it was explicitly reported, 52.4% (n = 11) required participants to engage with an exercise or behavioural programme as part of their use of the wearable device [38, 42–44, 46, 48, 54, 55, 57, 63, 68].

### Usability assessment: quantitative methods

**Fig. 2:**
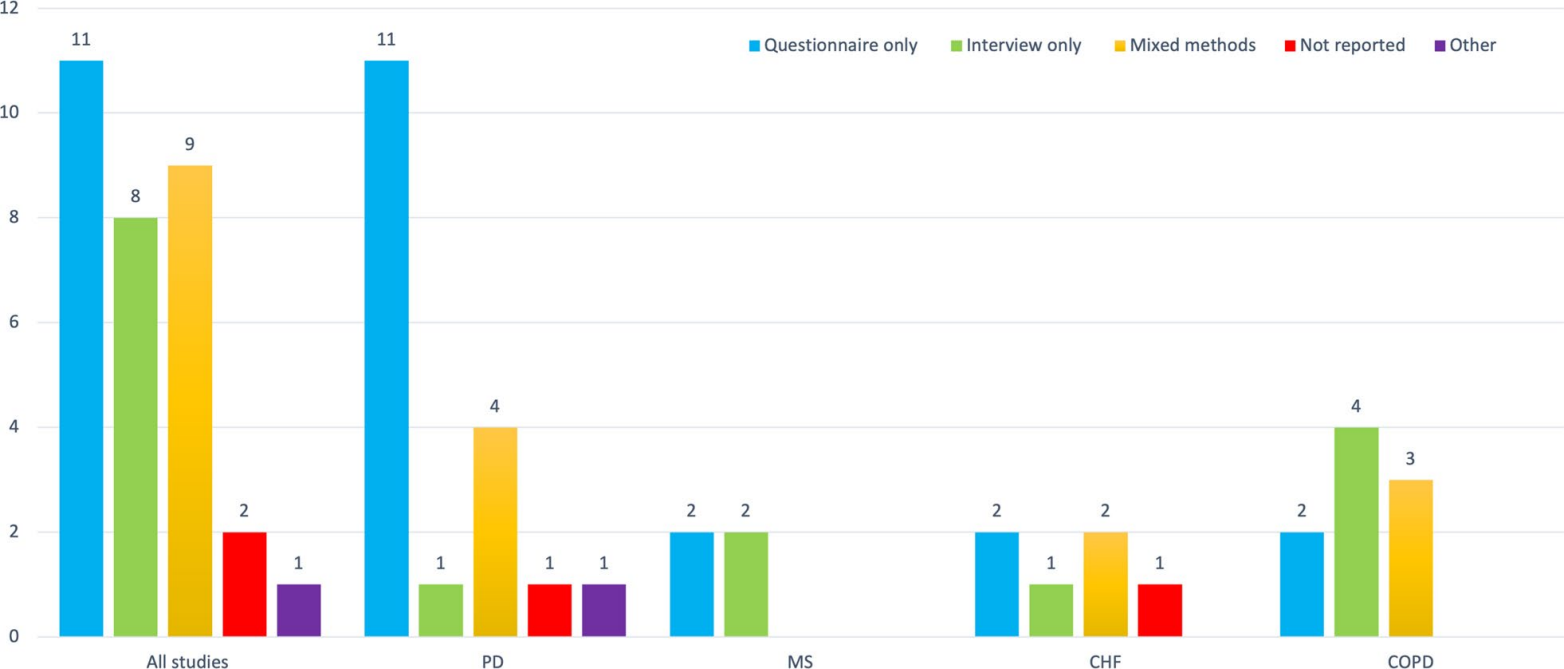
Methods to assess usability across all studies

The majority of studies used only quantitative methods to assess usability (Fig. 2), specifically questionnaires (45.9% n = 17) [34–37, 40, 43–45, 48, 49, 51–54, 57, 66, 69]. Eight studies used only qualitative methods such as interviews or focus groups (21.6%) [38, 41, 47, 55, 56, 65, 68, 70], a further nine implemented mixed methods (24.3%) [39, 42, 46, 50, 60, 62, 63, 65, 67], two studies failed to report what methods they used, yet reported usability findings in their results or conclusions (5.4%) [59, 61], while a single study used researcher observations and field notes to document participants words and perceived usability (2.7%) [58].

**Fig. 3:**
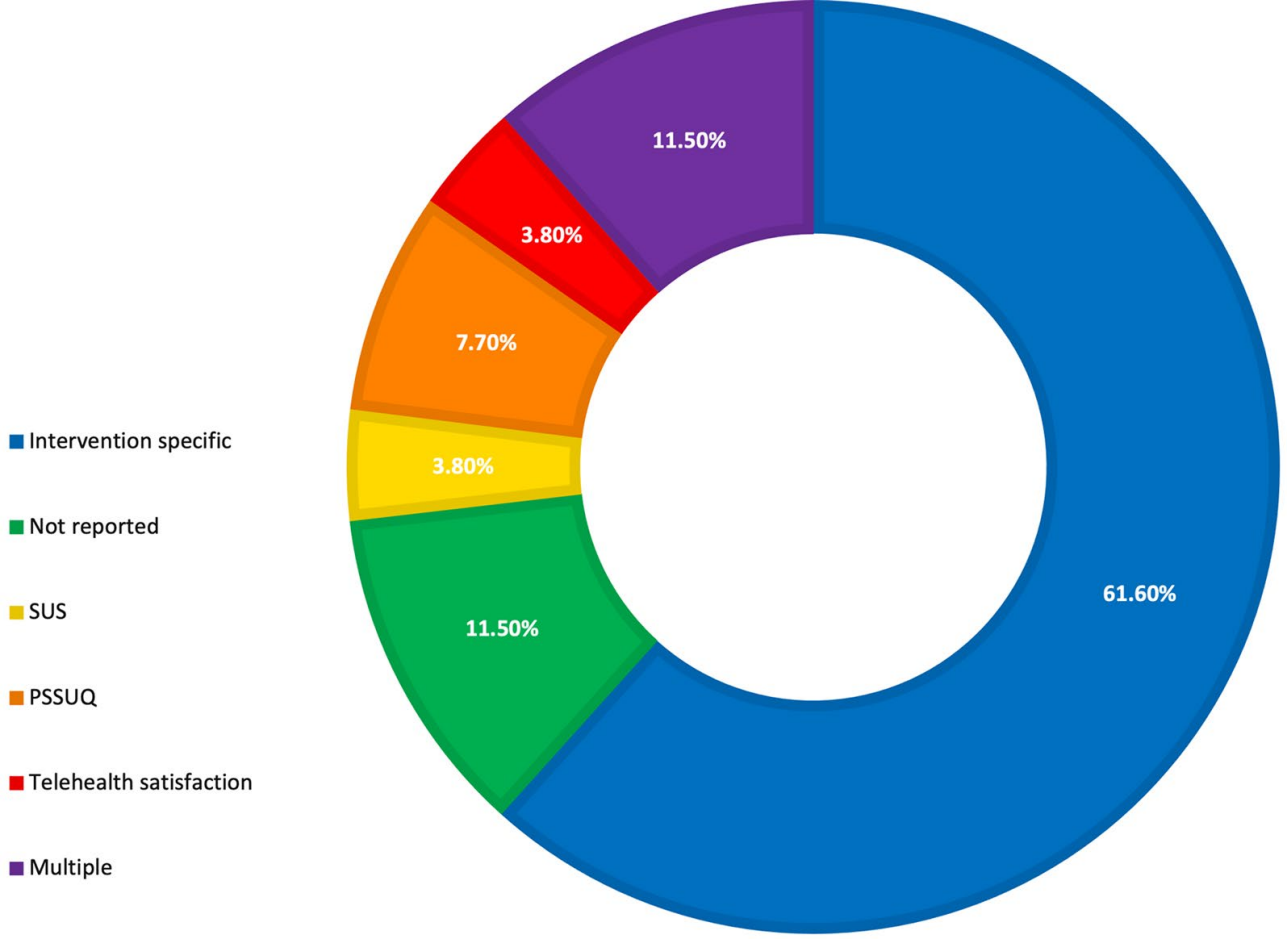
Questionnaries used to assess usability

Of the 26 studies that implemented questionnaires, (either individually or within mixed methods) 61.5% (n = 16) used an intervention specific questionnaire (Fig. 3) [35, 36, 39, 42–45, 48–54, 66, 69]. When reporting the content of their questionnaires (both previously validated and intervention specific), studies often listed the factors that were under consideration within their questionnaire, however just 10 studies (38.5%) provided the questions that they asked [34, 36, 37, 43, 48, 51, 53, 54, 66, 69]. In total 28 factors were listed as being examined as part of the questionnaires, across all 26 studies, including comfort, learnability, helpfulness and satisfaction (Additional file 1: Table S1).

Thirteen studies (50.0%) failed to list an overall result or score for their questionnaires [34, 40, 42, 43, 49–53, 60, 63, 65, 69]. For those 13 that did report a score, an overall average was assigned to 38.5% of studies (n = 5) [35, 39, 44, 45, 54], while 15.4% listed their results as a percentage score (n = 2) [37, 57]. An average result per question was listed for 23.1% of studies (n = 3) [46, 48, 66], while a further 23.1% of studies listed an average per questionnaire subsection (n = 3) [35, 62, 67]. One study listed the raw data of responses (7.7%) [36].

### Usability assessment: qualitative method

**Fig. 4:**
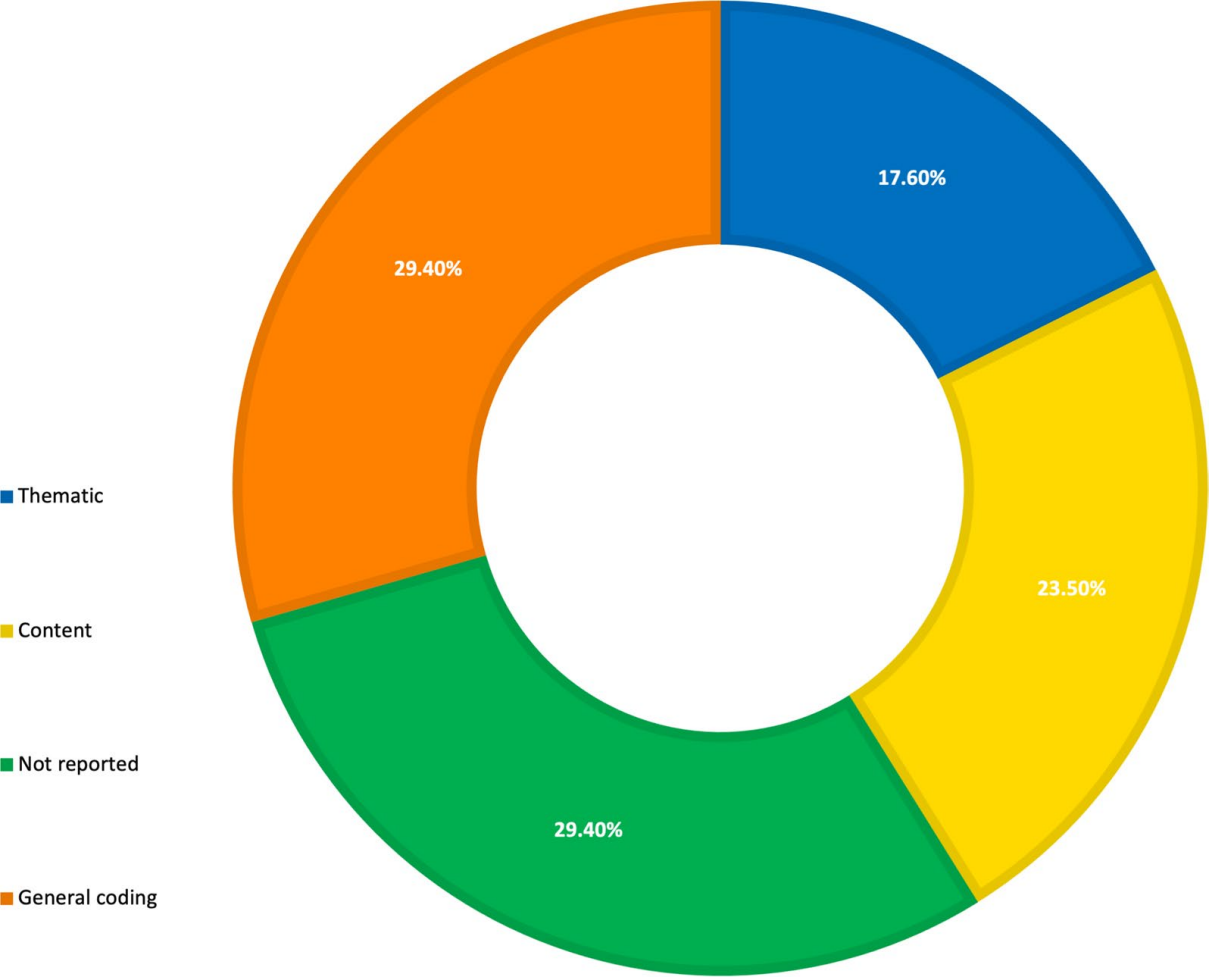
Analysis methods for qualitative components

Of the 17 studies that listed qualitative methods of assessment, six failed to report any method of analysis (35.3%) [38, 39, 58, 60, 63, 67]. A further five appeared to code generally rather than using any recognised formal analysis method (29.4%) [42, 55, 56, 65, 70]. The remaining studies used either a thematic (17.6%; n = 3) [41, 47, 68] or content (17.6%; n = 3) [50, 62, 65] analysis (Fig. 4).

Limited information was provided regarding the qualitative methods. Of the potential 32 items listed on the COREQ checklist, the included studies in this review recorded an average of just 6.7 (5.5) of these items. Interview guides were not provided for studies, however much like the quantitative components, a number of factors were listed across all studies. Specifically, 16 different factors were examined within the qualitative components, including overall experience, difficulties faced, acceptance and comfort (Additional file 1: Table S1).

As a result of the heterogeneity of presented data and the poor methods of reporting, it was not possible to compare usability results either between or within quantitative and qualitative methods, or across cohorts. In response to this, the results as reported within each study were used to determine whether positive or negative statements were provided. A list of the reported results is provided in Table 5.

**Table 5:** List of positive and negative results in both the quantitative and qualitative usability assessments per cohort

Factor	Positive result, quantitatively (n; %)	Negative result, quantitatively (n; %)	Positive result, qualitatively (n; %)	Negative result, qualitatively (n; %)
Parkinson’s Disease	(n = 15)	(n = 15)	(n = 5)	(n = 5)
Willingness to continue (with device or programme)	7 (46.7%)	1 (6.7%)	1 (20.0%)	
Easy to use	5 (33.3%)	1 (6.7%)	1 (20.0%)	
Satisfied (with device or programme)	5 (33.3%)			
Acceptable	3 (20.0%)			
User friendly	2 (13.3%)			
Comfort/Pain	4 (26.7%)		2 (40.0%)	
Supports self-management of condition	1 (6.7%)			
Tolerable levels of engagement	2 (13.3%)			
No interference in activities	2 (13.3%)			
Positive experience overall	2 (13.3%)			
No harm	2 (13.3%)		1 (20.0%)	
Would recommend to others	2 (13.3%)			
Motivational			2 (40.0%)	
Prefers digital to self-report/face-to-face	1 (6.7%)			1 (20.0%)
Reliable	1 (6.7%)			
Beneficial	1 (6.7%)		1 (20.0%)	
Increased awareness of behaviour			1 (20.0%)	
Technical literacy		2 (13.3%)		
Technical issues				1 (20.0%)
Unsecure attachment		1 (6.7%)		1 (20.0%)
Self-conscious while wearing		2 (13.3%)		2 (40.0%)
Altered behaviour		2 (13.3%)		
Burden		2 (13.3%)		
Irritation		1 (6.7%)		
Useful		1 (6.7%)		
Privacy concerns		1 (6.7%)		1 (20.0%)
Needed help/support to use		1 (6.7%)		
Waterproof				1 (20.0%)

MS	(n = 2)	(n = 2)	(n = 2)	(n = 2)
Willingness to continue (with device or programme)	1 (50.0%)			
Acceptable	1 (50.0%)			
Comfort/Pain	1 (50.0%)			
Tolerable levels of engagement	1 (50.0%)			
Increased awareness of behaviour			2 (100%)	
Valued feedback			1 (50.0%)	
Motivational			1 (50.0%)	
Desire for more (feedback)			2 (100%)	
Supports self-management of condition			1 (50.0%)	
Feedback not personalised/disease specific				2 (100%)
Burden				1 (50.0%)
Needed help/support to use				1 (50.0%)
Device loss				1 (50.0%)

CHF	(n = 5)	(n = 5)	(n = 7)	(n = 7)
Willingness to continue (with device or programme)	1 (20.0%)			1 (%)
Easy to use	3 (60.0%)	1 (20.0%)	1 (%)	
Enjoyed using			1 (%)	
Satisfied	1 (20.0%)			
User friendly	1 (20.0%)			
Supports self-management of condition	2 (40.0%)			
Motivational	1 (20.0%)			1 (%)
Forgot to use/sync		1 (20.0%)		1 (%)
Technical issues		1 (20.0%)		2 (%)
Needed help/support to use				1 (%)

COPD	(n = 4)	(n = 4)	(n = 3)	(n = 3)
Willingness to continue (with device or programme)	1 (25.0%)			
Easy to use	1 (25.0%)	2 (50.0%)	2 (66.7%)	
Comfort/Pain			1 (33.3%)	2 (66.7%)
Increased awareness of behaviour			2 (66.7%)	
Desire for more feedback			1 (33.3%)	
Satisfied	2 (50.0%)			
User friendly	2 (50.0%)			
Beneficial			1 (33.3%)	
Acceptable	2 (50.0%)			
Altered behaviour			1 (33.3%)	
N issues encountered			3 (100%)	
Valued feedback			2 (66.7%)	
Motivation	1 (25.0%)		1 (33.3%)	
Enjoyed	1 (25.0%)			
Unsecure attachment				2 (66.7%)
Adverse events		1 (25.0%)		1 (33.3%)
Irritation		1 (25.0%)		1 (33.3%)
Technical issues		1 (25.0%)		1 (33.3%)
Technical literacy		1 (25.0%)		1 (33.3%)
Feedback not personalised/disease specific		1 (25.0%)		1 (33.3%)
Privacy				1 (33.3%)
Burden				2 (66.7%)
Disconnect between perceived activity and feedback				2 (66.7%)
Battery				1 (33.3%)
Bulky				2 (66.7%)
Data upload issues				2 (66.7%)
Needed help/support to use				1 (33.3%)

### Usability findings

It was not possible to conduct an assessment of the usability findings.

### Study quality

The COREQ quality results have been documented in the “Usability assessment: qualitative method” section. Table 6 outlines the results from the AXIS quality tool. Six studies did not report or had unclear reporting of more than half of the 20-items on the list [38, 39, 59–61, 63]. The items that were most poorly reported across studies were; was the sample size justified, was the selection process likely to select participants that were representative of the target population, were measures taken to address and categorise non-responders and, does the response rate raise concerns about non-response bias.

**Table 6:** AXIS quality tool for cross-sectional studies

Study	1	2	3	4	5	6	7	8	9	10	11	12	13	14	15	16	17	18	19	20
Adams et al.	+	+	−	+	+	−	−	−	−	−	−	+	?	N/A	+	+	+	+	+	+
Albani et al.	+	+	−	+	−	−	−	+	+	N/A	+	−	?	N/A	+	+	+	−	−	+
Bachlin et al.	+	+	−	+	−	−	?	+	−	N/A	+	+	?	N/A	+	+	+	+	-	+
Botros et al.	+	+	−	+	−	−	−	+	+	+	+	−	?	N/A	+	+	+	+	−	+
Cancela et al.	?	?	−	+	−	−	−	−	−	N/A	−	−	?	N/A	+	+	+	−	−	+
Cancela et al.	?	?	−	+	−	−	−	+	+	N/A	+	−	?	N/A	+	+	+	−	−	+
Carpinella et al.	+	+	−	+	+	?	+	+	+	+	+	+	−	+	+	+	+	+	−	+
Chiauzzi et al.	+	+	−	+	−	−	−	+	−	N/A	+	+	−	−	+	+	+	+	−	+
Colon-Semenza et al.	+	+	−	+	+	+	+	+	−	N/A	+	+	−	−	+	+	+	+	−	+
Deka et al.	+	+	+	+	+	?	−	+	+	N/A	−	−	?	N/A	+	+	+	−	−	+
Ellis et al.	+	+	−	+	+	−	+	+	+	+	+	+	?	N/A	+	+	+	+	−	+
Ellis et al.	+	+	−	+	?	?	+	+	+	+	+	+	−	+	+	+	+	+	−	+
Ferreira et al.	?	?	−	+	?	−	−	+	+	+	+	+	?	N/A	+	+	+	−	+	+
Floegel et al.	+	+	−	+	?	?	+	+	+	N/A	+	+	−	+	+	+	+	+	−	+
Ginis et al.	+	+	−	+	?	−	+	+	+	+	+	+	−	+	+	+	+	+	+	+
Heijmans et al.	+	+	−	+	−	−	−	+	?	N/A	−	+	?	N/A	+	+	+	+	−	+
Hermanns et al.	+	+	−	+	−	−	−	+	+	N/A	+	+	?	N/A	+	−	+	+	−	−
Joshi et al.	?	?	−	+	?	−	−	+	+	N/A	+	+	?	N/A	+	+	+	+	+	+
KaY et al.	+	+	+	+	+	+	+	+	+	+	−	+	−	N/A	+	+	+	+	−	+
McNamara et al.	+	+	−	+	+	+	−	+	−	+	+	+	?	N/A	+	+	+	+	−	+
Midaglia et al.	+	+	−	+	+	−	−	+	−	+	+	+	?	N/A	+	+	+	+	+	+
Moy et al.	+	+	−	+	+	−	−	+	+	+	+	+	?	N/A	+	+	+	+	−	+
Orme et al.	+	+	−	+	+	+	+	+	+	+	+	+	−	+	+	+	+	+	−	+
Silva de Lima et al.	+	+	−	+	+	+	+	+	+	+	+	+	−	+	−	−	+	+	−	+
Stack et al.	+	+	−	+	+	−	+	+	−	N/A	−	+	−	+	+	+	+	+	−	+
Strisland et al.	−	−	−	+	−	−	−	−	−	−	−	−	−	−	−	−	+	−	−	−
Svagard et al.	+	−	−	+	−	−	−	−	−	−	−	−	−	−	−	−	−	−	−	−
Tzallas et al.	−	?	−	−	−	−	−	+	+	N/A	−	−	?	N/A	N	+	+	−	−	−
van der Weegen et al.	+	+	−	+	−	−	−	+	+	N/A	+	+	?	N/A	+	+	+	+	?	−
Varnfield et al.	+	+	−	+	?	−	−	?	?	N/A	−	−	?	N/A	?	+	−	−	−	?
Verwey et al.	+	+	−	+	+	−	−	+	+	+	−	+	?	N/A	+	+	+	−	−	+
Verwey et al.	+	+	−	+	+	+	+	+	+	+	+	+	−	+	+	+	+	+	−	+
Vooijs et al.	+	+	−	−	?	−	−	+	+	+	+	+	?	N/A	+	+	+	+	−	−
Vorrink et al.	+	+	−	+	−	−	−	+	+	+	+	+	?	N/A	+	+	+	+	−	−
Wendrich et al.	+	+	−	+	?	−	−	+	+	N/A	+	+	−	N/A	+	+	+	+	+	+
Werhahn et al.	+	+	−	+	+	+	−	+	+	+	+	+	?	N/A	+	+	+	+	−	+
Wu et al.	+	+	−	+	+	+	+	+	−	N/A	+	+	?	N/A	+	+	+	+	−	+

1: Were the aims and objectives of the study clear

2: Was the study design appropriate for the stated aims

3: Was the sample size justified

4: Was the target/reference population clearly defined

5: Was the sample frame taken from an appropriate population base so that is closely represented the target population under investigation

6: Was the selection process likely to select participants that were representative of the target population

7: Were measures taken to address and categorise non-responders

8: Were the risk factor and outcome variables measured appropriate to the study aims

9: Were the risk factor and outcome variables measured correctly using instruments that had been trialled piloted or published previously

10: Is it clear what was used to determine statistical significance

11: Were the methods sufficiently described to enable them be repeated

12: Were the basic data adequately described

13: Does the response rate raise concerns about non-response bias

14: If appropriate was information on non-responders provided

15: Were results internally consistent

16: Were the results presented for analysis described in the methods

17: Were the authors’ discussions and conclusions justified by the results

18: Were the limitations of the study discussed

19: Were there any funding sources or conflicts of interest that may affect the authors’ interpretation of results

20: Was ethical approval or consent of participants obtained

## Discussion

This systematic reviewed aimed to explore the usability of wearable devices to measure gait and physical activity in a range of cohorts with chronic health conditions. However, due to the heterogeneity in how usability is measured, combined with consistently poor reporting in the included studies, this aim was not able to be achieved. Ultimately this is the result of a poor quality body of literature. Researchers that include usability assessments within their study designs, are either not completing these assessments to an acceptable standard, or are failing to adequately report them. Although this points to a wider issue with how usability is defined, either way, at a time when research transparency is critical, and when supplemental files are commonly used, poor, basic reporting cannot be considered acceptable. Significant improvements in how usability research is defined and conducted is required to truly understand this concept, and as a result of this review, some basic recommendations for the same can be made.

The use of wearables within research is still relatively new, as evidenced by the years of publication both within this study and as reported elsewhere [72]. It is therefore understandable that just 24% of the studies within this review focused specifically on usability, as much research to date has focused on the technical side of these devices. Indeed, similar research has also noted a lack of usability assessments for wearable devices, suggesting that this is a general issues amongst clinical conditions [73]. For wearables to realise their full potential however, researchers must now begin to focus more attention towards the human factors [74–76]. Admittedly, the wide focus of study aims included in this review opened the potential for studies for which usability wasn’t the priority of the assessment. However it nonetheless demonstrated that researchers are attempting to assess usability, but that the quality of these evaluations are, for the most part, not fit for purpose [74, 76], and in some cases amount to little more than a ‘tick the box’ exercise. Poorly communicated, incomparable results have been previously highlighted as a key issue in this domain [4]. Indeed, despite the relative modernity of this concept, usability is consistently highlighted as a necessary step in technological developments, and is included in both WHO and ISO guidance [1, 3]. However, it has not been impactful, with some suggesting that as a construct, usability is vague, not fit for purpose and is at a dead-end in terms of its potential for impact [77]. It is argued that the lack of theory underpinning usability has created an umbrella term in which pragmatism takes preference, with a focus on immediate, study specific concerns that will change depending in the study or product in question [77]. For instance, the usability needs of an app-based intervention that incorporates a wearable device for long-term use, may be very different than the clinical needs of a device intended for 24 h monitoring.

One of the criticisms of usability is that, as an umbrella term existing in a multi-disciplinary space, it spans too many factors and means different things for different people [4, 77]. Usability has been shown to span a range of concepts including comfort, safety, durability, reliability, aesthetics and engagement [76], all of which were covered in the questionnaires and interviews in this review. Although these aspects are relevant to whether a device is acceptable and will be used, they were rarely defined. Rather than attempt to group factors together, a decision was taken to report these factors in the same manner as the studies themselves did so as to highlight the heterogeneity in terminology. This lack of consistency may be partially explained by the absence of frameworks or theories to design usability assessments [4, 72]. Without clearly defined components or theoretical rationale, reasons for evaluating factors may be unclear, thus making comparisons and conclusions difficult. Usability research should learn from behaviour change research which has designed taxonomies of intervention content in order to improve reporting, transparency and understanding [78]. Indeed a call to arms has been made for a flexible, adaptive framework that bridges the communication gap between multiple professions and domains, and serves as a reporting tool for anyone looking to conduct usability research [4]. In the absence of such a tool however, researchers conducting usability assessments need to ensure that, at the very least, they describe their own rationale and content in full so as to begin to improve communication standards themselves.

The lack of consistency in usability terminology was not the only issue encountered within this review. Many basic aspects of study methodology were often not reported, including where the device was worn, how it was attached and how long it was worn for. Linked to this was a concerning lack of both questionnaires and interview guides that were used in usability assessments. It is simply not possible to conclude whether the results of a study are internally consistent unless the measurement tools used to derive conclusions are also supplied, and a failure to provide this information should not be considered acceptable [79, 80]. The suggested taxonomy or meta-framework of usability will not work as a reporting guideline, if existing guidelines are not even enforced by publishers. Future research, and publishing bodies, should ensure that full protocols and methods of assessment are included as part of the basic reporting requirements for publication, through use of supplemental files if required.

User-centred design processes may appear onerous to researchers who are not familiar with them, or for whom feel that usability is not the primary focus of their research (e.g. within pilot or feasibility trials). However, in line with the highlighted need for a better understanding of usability a concept, future usability research needs to also become patient-centred rather than study-centred to fully understand how devices are accepted and used across a variety of contexts and cohorts and to iterate devices in response to this [74]. Various methods to conduct this exist, including think aloud processes, questionnaires, and interviews. Specifically though, mixed methods should be considered gold standard as the sum of the qualitative and quantitative components provides more insight than either method can alone [81]. Mixed methods were used in less than a third of papers of this review, with a focus instead on quantitative methods alone, a finding that was also reported elsewhere [72]. Linked to this is a reliance on intervention specific questionnaires. Various validated measures of usability for wearable technology exist [82–84], yet these were infrequently used. Although there is a place for intervention specific measures for understanding specific study details [85, 86], when used alone they limit the ability to compare findings both across and within patient cohorts. Thus, future usability research should not only implement mixed methods as standard practice, but also combine intervention specific measures with previously validated questionnaire to allow for comparison and to derive both specific and generalisable insights.

This review was limited by its inability to make definitive conclusions as a result of the heterogeneity found in almost every variable considered. Furthermore, the focus on five specific disease cohorts limits the generalisability of results, although given the context specific nature of usability, generalisations would not be recommended regardless of what findings were derived from the review. However, the selected cohorts nonetheless provide examples of a variety of conditions which is a progression beyond the typical focus of healthy adults. Furthermore, this review focused only on wearables that measure gait and physical activity and therefore inferences on other more general devices (e.g. blood pressure, heart rate etc.) cannot be made. Finally, it was sometimes difficult to separate whether the usability findings related specifically to the wearable or to the overall system that was being evaluated. However, given that 83.8% of the wearables in this review were paired with another device, this is perhaps not concerning as participants themselves will experience the wearable as part of this system and so their feedback will always intrinsically link the two together.

## Conclusion

Technology is at an unprecedented point in its development as it has the potential to drastically alter both how we evaluate and treat various healthcare conditions. However, this can only be realised if it is widely adopted by all stakeholders, including patients and participants. It is possible to infer that wearable devices for gait and physical activity are both acceptable and usable, given the wide variety of devices, placements, durations of use etc., that were reported in this review. However, usability, and patient-centred design, is a critical component of any intervention. It is therefore not enough to simply infer usability. While a call to arms has been made for reporting guidelines and hierarchical frameworks of usability, until they are developed researchers need to be aware of the current pitfalls of the term, and work where possible to avoid them. As such, in the absence of being able to make specific usability conclusions, this review instead recommends that future research needs to:

1. Conduct usability assessments as standard, irrespective of the cohort under investigation or the type of study undertaken.
2. Adhere to standard reporting standards (e.g. COREQ) including the basic details of the study. Full copies of any questionnaires and interview guides should be supplied through supplemental files.
3. Utilise mixed methods research to gather a more comprehensive understanding of usability than either qualitative or quantitative research alone will provide.
4. Use previously validated questionnaires alongside any intervention specific measures.
5. Consider the learnings and insights that can be gained from usability research from multiple domains.

## Supplementary Information

**Additional file 1: Table S1.** List of factors assessed in both the quantitative and qualitative methods of usability assessments

## Data Availability

The datasets used and/or analysed during the current study are available from the corresponding author on reasonable request.

## Abbreviations

CHF: Congestive heart failure
COPD: Chronic obstructive pulmonary disorder
MS: Multiple sclerosis
PD: Parkinson’s disease
PFF: Proximal femoral fracture
