# Supplementary material for: Assessing the usability of wearable devices to measure gait and physical activity in chronic conditions: a systematic review
Source: J Neuroeng Rehabil. 2021 Sep 15;18:138. doi: 10.1186/s12984-021-00931-2 (PMC8444467; doi:10.1186/s12984-021-00931-2)
Supplement: Supplementary file 1 — Additional file 1: Table S1. List of factors assessed in both the quantitative and qualitative methods of usability assessments [file 12984_2021_931_MOESM1_ESM.docx]

Supplemental file 1

**Table: List of factors assessed in both the quantitative and qualitative methods of usability assessments**

| **Factor** | **Quantitative**  **(number of studies; % of studies with quant methods)** | **Qualitative**  **(number of studies; % of studies with qual methods)** |
| --- | --- | --- |
| Ease of use | 8 (30.8%) |  |
| Comfort | 8 (30.8%) | 1 (6.3%) |
| Satisfaction | 7 (26.9%) | 4 (25.0%) |
| Effectiveness (of programme or device) | 5 (19.2%) |  |
| Overall experience | 4 (15.4%) | 6 (37.5%) |
| Willingness to continue (with programme or device) | 3 (11.5%) | 1 (6.3%) |
| Adequacy of information | 3 (11.5%) |  |
| Learnability | 3 (11.5%) |  |
| Difficulty/challenges experienced | 3 (11.5%) | 4 (25.0%) |
| Interference with daily life/activities | 2 (7.7%) |  |
| User interface | 2 (7.7%) |  |
| Simplicity | 2 (7.7%) |  |
| Pain | 2 (7.7%) |  |
| Exertion | 2 (7.7%) |  |
| Usability | 2 (7.7%) | 1 (6.3%) |
| Acceptance | 1 (3.8%) | 2 (12.5%) |
| Device attachment | 1 (3.8%) |  |
| Benefit | 1 (3.8%) |  |
| Likability (of mentor) | 1 (3.8%) |  |
| User friendliness | 1 (3.8%) |  |
| Adverse events | 1 (3.8%) |  |
| Donning/doffing | 1 (3.8%) |  |
| Efficiency | 1 (3.8%) |  |
| Affect | 1 (3.8%) |  |
| Helpfulness | 1 (3.8%) |  |
| Control | 1 (3.8%) |  |
| Usefulness | 1 (3.8%) |  |
| Feasibility | 1 (3.8%) |  |
| Feedback |  | 3 (18.8%) |
| Concerns |  | 2 (12.5%) |
| Medical changes to condition |  | 2 (12.5%) |
| Functionality |  | 2 (12.5%) |
| Future use of device |  | 2 (12.5%) |
| Improvements needed |  | 2 (12.5%) |
| Demand |  | 1 (6.3%) |
| Managing data |  | 1 (6.3%) |
| Compliance |  | 1 (6.3%) |
